# MRI assessment of glutamine uptake correlates with the distribution of glutamine transporters and cancer stem cell markers

**DOI:** 10.1038/s41598-022-09529-7

**Published:** 2022-04-01

**Authors:** Yoojeong Seo, Joyeon Kang, Tae Il Kim, Chan Gyu Joo

**Affiliations:** 1 Department of Internal Medicine and Institute of Gastroenterology, Severance Hospital, Yonsei University College of Medicine, 50-1 Yonsei-ro, Seodaemun-gu, Seoul, 03722 Republic of Korea; 2 Yonsei Cancer Prevention Center, Severance Hospital, Yonsei University College of Medicine, Seoul, Republic of Korea; 3 Graduate School of Medical Science, Brain Korea 21 Project, Yonsei University College of Medicine, Seoul, Republic of Korea; 4 Severance Biomedical Science Institute, College of Medicine, Yonsei University, 50-1 Yonsei-ro Seodaemun-gu, Seoul, 03722 Republic of Korea

**Keywords:** Cancer imaging, Cancer metabolism

## Abstract

Glutamine provides carbon and nitrogen for macromolecular synthesis and participates in adenosine triphosphate (ATP) generation, anabolic metabolism, redox homeostasis, cell signaling, and cancer stem cell (CSC) metabolism. New treatment strategies targeting glutamine metabolism in cancer have emerged recently. We previously reported the magnetic resonance imaging (MRI) assessment of glutamine uptake by tumors and activated glutamine metabolism in CSC. In the present study, using MRI, we determined the correlation between glutamine uptake and the distribution of glutamine transporters, namely ASCT2 and SLC38A2 (SNAT2), glutaminase (GLS), and CSC markers, such as CD44 and CD166, in a mouse xenograft model of HT29 human colorectal cancer cells. MRI data revealed an obvious change in intensity following glutamine administration. Immunohistochemistry (IHC) results indicated that ASCT2 staining was stronger in regions that exhibited high glutamine uptake (p = 0.0079). Significant differences were found in the IHC staining intensities of SNAT2, GLS, and CSC markers in the areas of high and low glutamine uptake (p = 0.0079, p = 0.0159 and p = 0.0079, respectively). We also investigated the effect of an ASCT2 inhibitor on the uptake of glutamine using MRI. A statistically significant difference in the initial glutamine uptake was found after ASCT2 inhibitor administration. To conclude, glutamine uptake is positively correlated with the distribution of ASCT2 and certain CSC markers.

## Introduction

Cancer cells can reprogram their glucose metabolism to achieve rapid proliferation, growth, and maintenance of their redox balance^1^. In normal cells, in the presence of oxygen, glucose is metabolized to pyruvate, which is then converted to acetyl-CoA in mitochondria and participates in the tricarboxylic acid (TCA) cycle, generating adenosine triphosphate (ATP). However, one of the major metabolic alterations in cancer cells involves aerobic glycolysis, in which pyruvate is converted directly to lactate, generating less ATP than oxidative phosphorylation in normal cells. In addition, cancer cells tend to rely on various alternative fuels, such as amino acids and lipids, to support their fast growth. An important amino acid utilized by cancer cells is glutamine^2,3^. Glutamine is the most abundant amino acid in plasma and an important energy source for cells both in normal and cancerous tissues^4^. It participates in ATP generation, redox homeostasis, and cell signaling in cancer cells.

Glutamine is transported into cells through the solute carrier family 1 neutral amino acid transporter member 5 (SLC1A5; also known as ASCT2)^5^. As its nitrogen is utilized in the synthesis of nucleotides and amino acids, glutamine contributes to biomass accumulation in cancer. Glutamine is also an important source of carbon in cancer and it provides an alternative way of synthesizing acetyl-CoA for fatty acid biosynthesis. Many glutamine transporters, such as solute carrier family 38a member 1 (SLC38A1, also known as SNAT1) and solute carrier family 38a member 2 (SLC38A2, also known as SNAT2), exist and are known to play an important role in tumorigenesis^6^. In addition, we previously demonstrated the relationship between ASCT2 and some cancer stem cell (CSC) populations, and the CSC-suppressing effect of an ASCT2 inhibitor^7^. Overall, as the role of glutamine in cancer has been revealed, new treatment strategies targeting glutamine metabolism have emerged.

In an effort to develop a non-invasive imaging method for assessing glutamine metabolism, we previously reported that magnetic resonance imaging (MRI) can be used to measure the initial uptake of glutamine in cancers^8^. In the present study, we further investigated how the glutamine MRI assessment reflects the biological aspects of cancers using a mouse xenograft model of the HT29 human colorectal cancer cell line.

**Figure 1:**
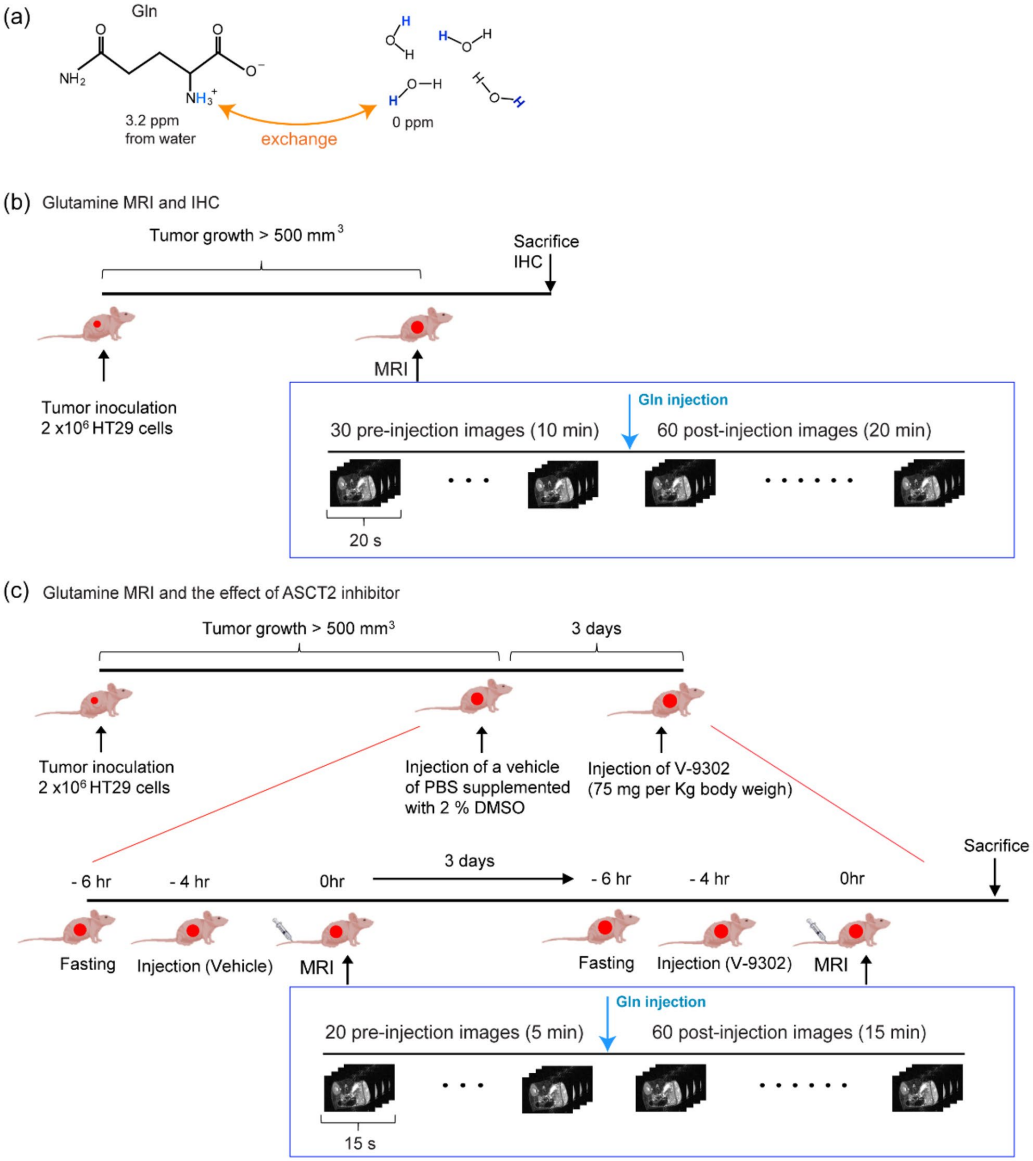
The principle of glutamine magnetic resonance imaging (MRI) and experimental design using an in vivo xenograft model. (**a**) The structure of glutamine. The amine protons of glutamine are exchanged with water protons. The chemical shift indicated here is in reference to the water signal. (**b**) Experimental design for investigating the correlation between MRI assessment of glutamine uptake and distribution of ASCT2 and CSC markers. (**c**) Experimental design for examining the effect of ASCT2 inhibitor V-9302 on MRI assessment of glutamine uptake.

In the glutamine MRI study, image contrast is generated by the chemical exchange between amine and water protons (Fig. 1a). This method relies on the fact that the magnetic properties of water protons are rendered via a chemical exchange process^9–13^. Particularly rendered, owing to the chemical shift difference and the fast exchange between amine and water protons, is the spin–spin relaxation time (T_2_) of water. Therefore, the presence of glutamine is observed as low intensity in T_2_-weighted images. In our previous study, the uptake of intravenously injected glutamine in cancer was measured using T_2_-weighted MRI, and it was found that glutamine uptake is spatially heterogeneous. However, whether the localized presence of glutamine measured using MRI genuinely reflects metabolic tumor heterogeneity or simply shows the well-perfused regions remains unknown. Therefore, in the present study, we investigated the correlation between MRI assessment of glutamine uptake and the expression of GLS, ASCT2, SNAT2, and CSC markers and effect of an inhibitor of the glutamine transporter ASCT2 on glutamine uptake in MRI.

## Results

### Experimental designs for the glutamine MRI study

In the glutamine MRI study, image contrast was generated by the chemical exchange between amine and water protons (Fig. 1a). We performed in vivo xenograft experiments to measure glutamine uptake in HT29 cells using MRI. Palpable tumors are typically visible within 2 weeks. For imaging studies, tumors were allowed to grow to approximately 500 mm^3^. Figure 1b,c show the experimental design of the glutamine MRI study and the effect of the ASCT2 inhibitor. No notable difference was observed in the tumor sizes and growth rates of male and female mice.

### Time-dependent changes in glutamine uptake intensity in xenograft tumors

**Figure 2:**
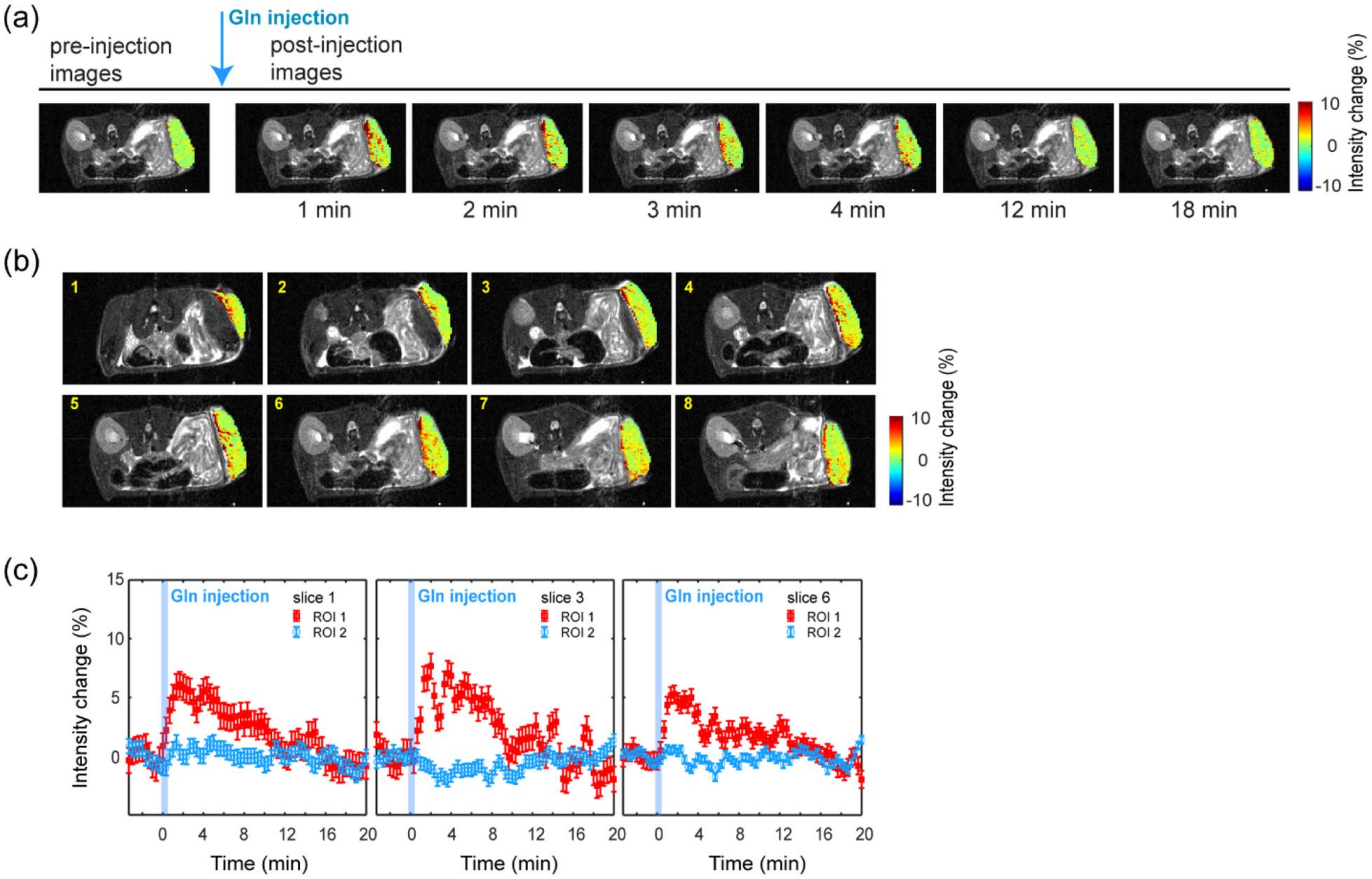
Images of glutamine uptake in the mouse xenograft model using HT29 cells. Only the intensity change in the tumor region is displayed. (**a**) Dynamic images from a single slice over time. (**b**) Representative images from serial slices of a single animal. The average of the intensity change over the first 4 min post injection is displayed. The slice number is indicated on the images. (**c**) Time-dependent intensity changes of T_2_ weighted images from the selected slices. Glutamine injection is indicated in blue. The time-dependent profiles are displayed for the regions of strong (ROI 1, indicated in red) and weak (ROI 2, indicated in blue) glutamine uptake.

**Figure 3:**
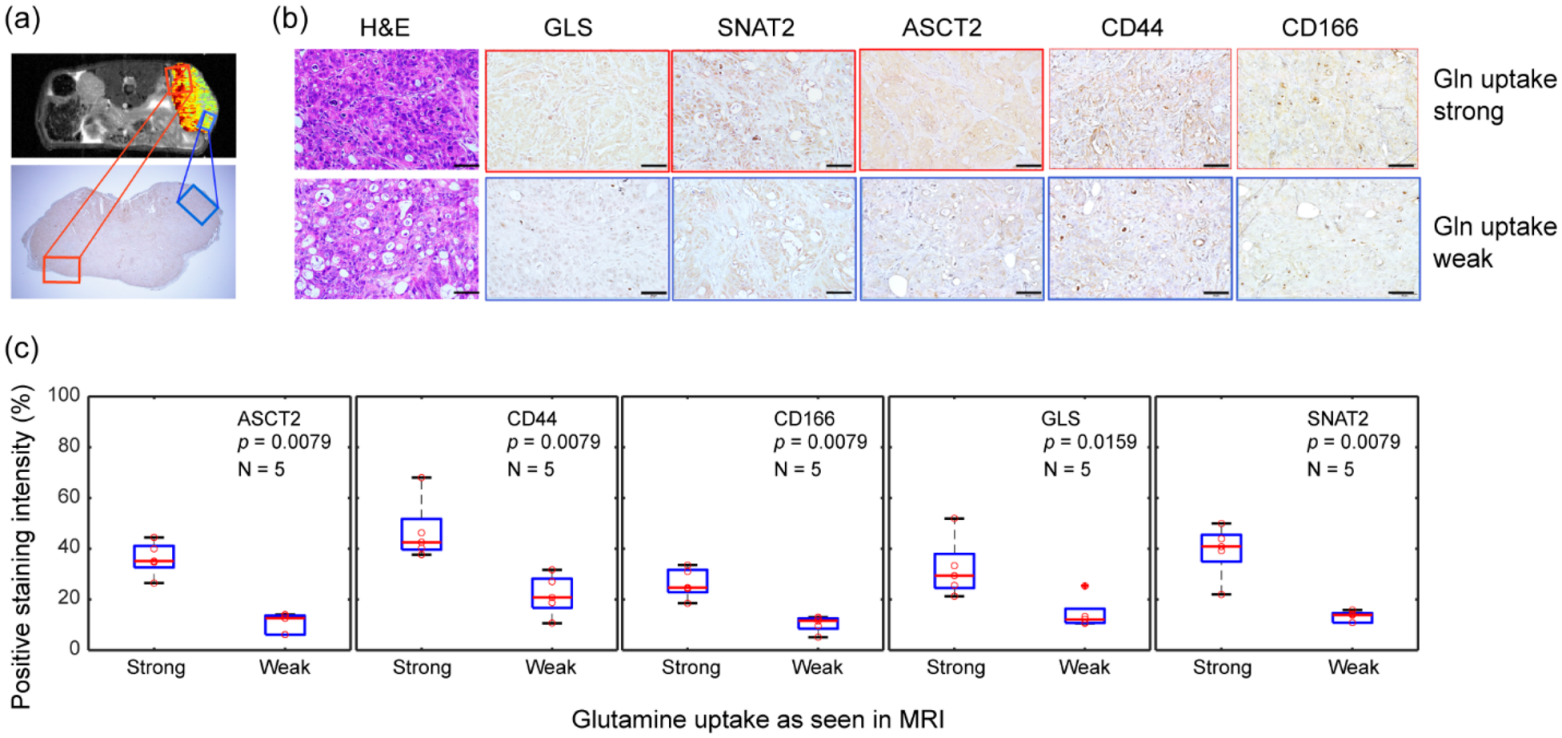
Correlation between glutamine uptake in magnetic resonance imaging (MRI) and the expression of ASCT2 and CSC markers. (**a**) The xenograft HT29 tumor, which showed both strong glutamine uptake (marked in red) and weak glutamine uptake (marked in blue) in the MRI image was sectioned to verify the MRI results. The image of the tumor below was obtained after IHC staining for ASCT2 expression. Scale bar = 2 mm. (**b**) IHC staining for ASCT2, GLS, SNAT2, CD44, and CD166 expression in tumor regions that show strong (top row, outlined in red) and weak (bottom row, outlined in blue) glutamine uptake. Scale bar = 50 μm (**c**) The *t*-test results of IHC scores (ASCT2, CD44, and CD166). (n = 5 biologically independent samples per group).

We measured the T_2_-weighted images using a spin-echo sequence with a 48-ms effective echo time while glutamine solution was injected intravenously. The intensity change was calculated using Eq. (1). Image contrast was generated from the differences in the T_2_ values of water (Fig. 2). The decrease in intensity caused by the presence of glutamine appeared as a positive ΔS(t). Figure 2a shows the representative dynamic images of a single slice obtained from a mouse model implanted with HT29 cells. The MRI intensity change is clearly visible following the injection of glutamine solution. Figure 2b shows images of adjacent slices across the tumor mass from a single mouse. Each slice was 1-mm-thick. Regions with strong and weak glutamine uptake across the tumor are distinguishable. Images from all mice are included in Supplementary Fig. 1. In Fig. 3c, the profile of the intensity change in three selective slices as a function of time is plotted for regions of strong (ROI 1) and weak (ROI 2) glutamine uptake. The glutamine signal was most intense in approximately the first 4 min. It then gradually diminished over time. Individual profiles varied depending on the mouse and also by region within the same tumor.

### Correlation between glutamine uptake in MRI and the expression of ASCT2 and CSC markers

To determine whether the expression of ASCT2, GLS, SNAT2, and CSC marker was strong in the area with high glutamine uptake and weak in the area with low glutamine uptake, the tumors were sectioned, and the tissue slices were selected by correlating with an MRI image showing the areas with weak and strong glutamine uptake. We next performed hematoxylin and eosin staining, and IHC staining of ASCT2, GLS, SNAT2, CD44, and CD166 (Supplementary Fig. 2). The IHC results are presented in Fig. 3. Regions with strong and weak glutamine uptake are outlined as red and blue, respectively, in Fig. 3a,b. We observed that the expression of ASCT2, GLS, SNAT2, and CSC markers (CD44 and CD166) was stronger in the areas with high glutamine uptake than in the areas with low glutamine uptake. Conversely, in areas with low glutamine uptake, the expression of ASCT2, GLS, SNAT2, and CSC markers was decreased. Figure 3c shows the positive staining intensity results for the IHC scores from five mice. The staining scores of ASCT2, GLS, and SNAT2 were higher in areas with strong glutamine uptake than in areas with weak glutamine uptake (p = 0.0079, p = 0.0159, and p = 0.0079, respectively). Significant differences were also observed in the IHC staining intensities of both CD44 and CD166 between areas of high and low glutamine uptake (p = 0.0079 and p = 0.0079, respectively).

### Decrease in glutamine uptake in MRI by pharmacological blockade of ASCT2 with V-9302

**Figure 4:**
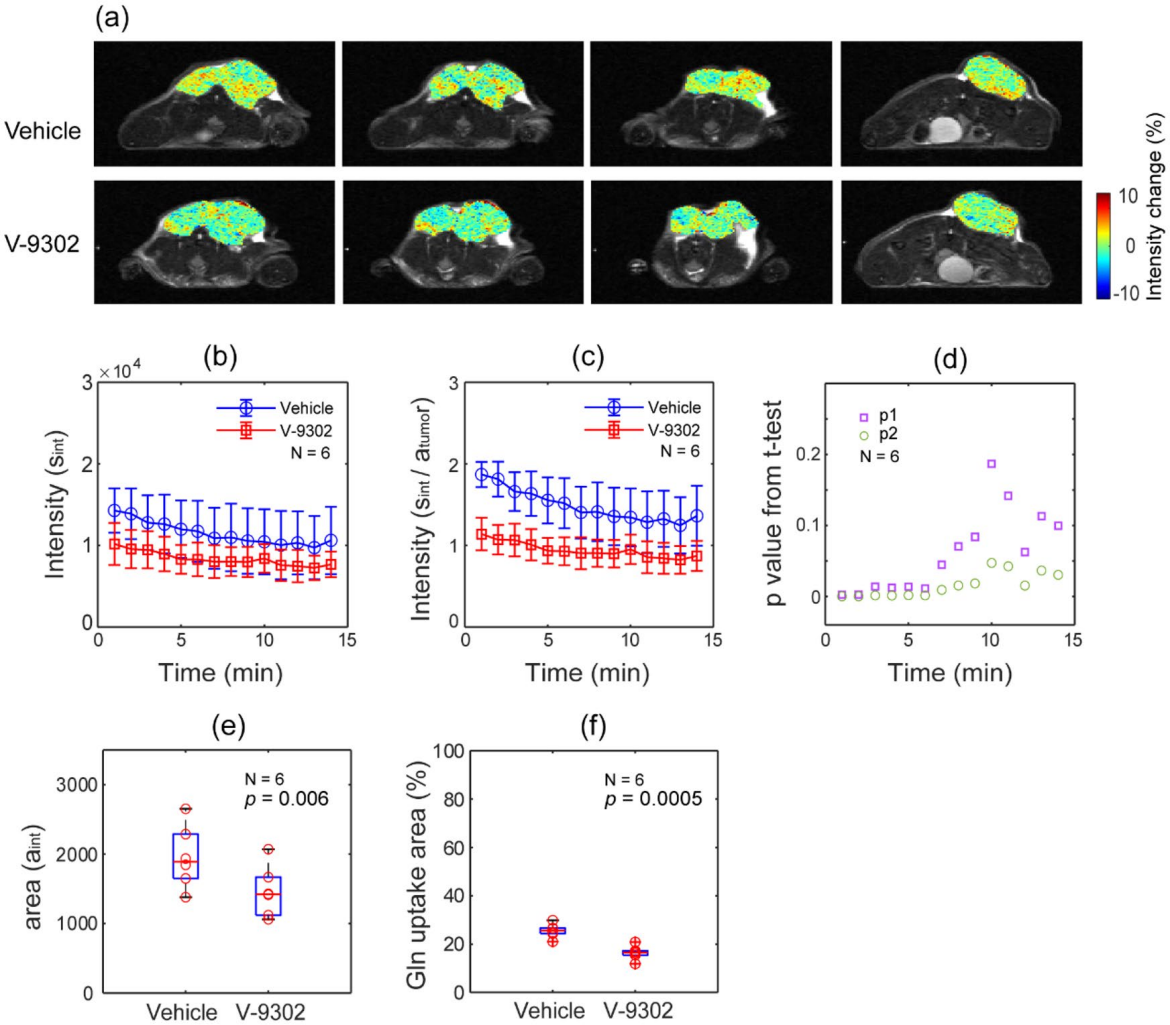
Decreased glutamine uptake in MRI due to the pharmacological inhibition of ASCT2 expression. (**a**) Comparison of representative glutamine MRI images (vehicle vs V-9302 treated) from a single mouse at a similar location. (**b**) The sum of the intensity of pixels that show a change higher than 4% (s_int_) is plotted as a function of post-injection time for all six mice. (**c**) Data in (**b**) were divided by the tumor size (a_tumor_) to account for the tumor growth during the 3 days between MRI scans. (**d**) p values (p1 from the *t*-test of s_int_ (**b**)) and p2 from the *t*-test of s_int_/a_tumor_ (**c**) are presented for the post-injection time. (**e**) The tumor area as a number of pixels that show a change higher than 4% (a_int_). It shows reduced glutamine uptake in V-9302-injected mice. (**f**) The relative area of a_int_ in the whole tumor, calculated using the formula (a_int_/a_tumor_) × 100.

To confirm the MRI assessment of the dynamic changes in glutamine uptake, we performed a glutamine MRI study after treatment with an ASCT2 inhibitor. The effect of the ASCT2 inhibitor on the glutamine uptake MRI is presented in Fig. 4. Figure 4a shows the representative images from similar areas of the same mouse. High intensity regions are visible in the images of vehicle-treated mice and not in the images of the V-9302-treated mice. Considering the signal-to-noise of the raw images, pixels that showed a change higher than 4% were selected, and then, the sum of intensities (s_int_) was calculated as a function of the post-injection time. Figure 4b shows the s_int_ of the images from vehicle- or V-9302-treated mice. Individual data are presented in Supplementary Fig. 3. To account for the tumor growth during the 3 days between the two MRI experiments, data in Fig. 4b were divided by tumor volume (a_tumor_, measured from MRI), and the results are shown in Fig. 4c.

To detect the initial uptake of glutamine, the tumor area with more than 4% intensity change (a_int_, counted as number of pixels) was calculated for the first 4 min following injection. The relative uptake area during the first 4 min was then calculated by comparing a_int_ to the whole tumor volume, a_tumor_. The p values obtained from the paired *t*-test performed on the data in Fig. 4b,c for each time point are presented in Fig. 4d as p1 and p2, respectively. A statistically significant change in the 6-min post glutamine injection time period was observed. The analysis of the number of pixels (Fig. 4e) and the relative area (Fig. 4f) with more than 4% intensity change was also statistically significant.

## Discussion

Recent studies have shown that both glucose and glutamine are used as energy sources in many types of cancers. Glutamine is imported into the cells via ASCT2 and other transporters. It then enters a complex metabolic network and promotes cell survival and growth, mainly with its involvement in the metabolic characteristics of CSCs^14^. In addition, ASCT2 overexpression is correlated with poor prognosis of patients with cancer and features of some CSCs^14^. Therefore, targeting glutamine metabolism pathways, including ASCT2, could represent a promising therapeutic strategy. Useful methods for identifying metabolic features of tumors, monitoring metabolic aspects of treatment responses, and imaging glutamine metabolism using a non-invasive approach might allow early detection of disease progression or response in terms of tumor glutamine metabolism. In the present study, we showed that glutamine-based molecular imaging by MRI could be used as a novel diagnostic tool to provide initial evidence of predictive and prognostic therapeutic response for anti-cancer drugs targeting glutaminolysis.

Regarding the principle of the glutamine MRI assessment, in the present study, the T_2_ change of water protons caused by the chemical exchange between glutamine amine protons and water protons was measured using a long TE spin-echo sequence. The difference in intensity generated by the presence of glutamine is determined by the relaxivity caused by exchange (R_2ex_). According to our previous study, the R_2ex_ of glutamine is 0.069 s^−1^ mM^−1^^8^. Glutamine is converted to glutamate in cancer cells. The chemical shift of amine protons in glutamate is separated from the water signal by approximately 3.2 ppm; therefore, glutamate also contributes to the change in water T_2_. The R_2ex_ of glutamate (0.102 s^−1^ mM^−1^) was higher than that of glutamine at physiological pH values. As reported in a hyperpolarized MRI study, the conversion from glutamine to glutamate is fast^14^. Therefore, it is reasonable to assume that the change in intensity shown in Fig. 2 was attributed to both the injected glutamine and the glutamate converted from glutamine. It should also be noted that extracellular glutamine may also contribute to this effect.

In terms of dynamic change and different distribution of glutamine uptake regions in MRI, Fig. 2b comprises representative post-injection images from a single mouse, showing the average change in intensity during the first 4 min after injection. Glutamine uptake occurred in all regions of the tumor, but in a rather localized way.

Furthermore, not only was the uptake spatially localized, but it also exhibited a different time profile, as shown by the time profiles from the representative slices presented in Fig. 3b for the regions of strong (ROI 1) and weak (ROI 2) glutamine uptake. In general, the uptake seen in ROI 1 increased immediately after injection and then gradually decreased over time. However, the pattern differed between individual regions and may have represented the different microenvironments or tumor cells with heterogeneous characteristics.

To determine the features of heterogeneous distribution of glutamine uptake regions, we compared the distribution of ASCT2, GLS, and SNAT2 with the glutamine uptake behavior revealed by MRI. IHC results revealed that the high ASCT2, GLS, and SNAT2 expression in the xenograft tumor was correlated with high glutamine uptake in the area. Glutamine MRI measured the initial uptake and the immediate conversion of glutamine, rather than the change in the endogenous metabolism. Therefore, the initial uptake correlated with the presence of ASCT2.

Moreover, to verify whether the localized signal of glutamine uptake was visible by perfusion difference through the tumor, we measured the change of glutamine uptake intensity in MRI after treatment with the ASCT2 inhibitor V-9302. As presented in Fig. 4, we observed a decrease in glutamine uptake due to suppressed ASCT2 expression. The MRI methods used in this work relied on the long TE to generate image contrast from chemical exchange. Considering the reduced signal-to-noise ratio because of long TE, we used pixels with a change above 4% for analysis. A statistically significant difference was present not only in the intensity magnitude but also in the number of pixels and the relative area of glutamine uptake. This finding implied that glutamine MRI does not only measures the well-perfused regions but also detects reduced glutamine uptake due to the ASCT2 inhibitor. Dynamic contrast enhanced (DCE) MRI can provide further insights on the effect of perfusion in glutamine MRI, although it is not included in our present study. It would also be useful to establish a kinetic uptake model of metabolites along with glutamine MRI, which will be discussed in future studies.

Regarding the biological significance of the ASCT2 inhibitor, Schulte et al. showed that the treatment with V-9302 in colorectal cancer (CRC) cell lines resulted in decreased mTOR activity, as assessed by investigating the expression of phosphor-S6 and phosphor-AKT (Ser473), which is consistent with diminished amino acid transportation and metabolism^15^.

Recent studies have shown that CSC markers might be predictors of cancer prognosis and are related to treatment resistance, recurrence, and metastasis of cancer. In CRC, CD44, CD166, CD133, and Lgr5 are considered typical CSC markers and are related to the Wnt/β-catenin signaling pathway^16^. In addition, Liao et al. reported that glutamine plays an important role in maintaining the stemness of cancer cells via a redox-mediated mechanism regulated by β-catenin^17^. We also showed that some CRC cells were relatively dependent on energy metabolism as an energy source, especially CSCs, and demonstrated high expression of ASCT2 in CSC population and the CSC-suppressing effect of the glutaminase (GLS) inhibitor^7^.

Therefore, in the present study, we substantiated the correlation between the glutamine uptake revealed by MRI and the presence of certain CSC markers (CD44 and CD166). CD44 also participates in redox regulation by stabilizing the subunit of xCT (the catalytic subunit of transport system x^−^_c_), exhibiting strong xCT activity. Thus, the correlation between glutamine uptake and CD44 may be related to the function of xCT, and in some cancers, glutamine addiction is explained by the functional coupling of xCT and ASCT2^18^. Glutamine taken into cancer cells via ASCT2 is converted to glutamate by GLS. Then, the xCT system exports glutamate from the cell in exchange for cystine to maintain the level of glutathione.

The metabolic heterogeneity of cancer is important because it may be related to the malignant transformation of cells, adaptation to different microenvironments, and early response to treatment. Recent preclinical studies have shown that targeting glutamine metabolism provides therapeutic opportunities^15,19^. Therefore, glutamine MRI may be used as a useful non-invasive imaging tool to assess metabolic changes during the development of new anti-cancer drugs. In addition, the situations in which PET-CT is not useful, that is, in some cancers where glucose is not used as an energy source when the tumor cell density is too low or the image background is strong, may be addressed by glutamine MRI. Importantly, the activity of ASCT2 and CSC markers can be quantitatively assessed indirectly using non-invasive glutamine MRI, which can detect changes in some stem cell markers in CSC-targeting therapies.

The present study demonstrated that MRI assessment of glutamine uptake could be used to assess the distribution of glutamine transporter ASCT2 and certain CSC markers. In addition, glutamine MRI could measure the reduction in glutamine uptake caused by an ASCT2 inhibitor. Based on our results, we believe that the glutamine MRI study may be useful for investigating the metabolic changes associated with therapies targeting glutaminolysis and CSCs.

## Methods

### Cell line and culture conditions

The HT29 colorectal cancer cell line was purchased from the American Type Culture Collection (Manassas, VA, USA). The cells were cultured in Dulbecco’s modified Eagle’s medium (DMEM; Invitrogen, Carlsbad, CA, USA) supplemented with 10% fetal bovine serum (Gibco, Franklin Lakes, NJ, USA) and 1% penicillin/streptomycin (Invitrogen) and incubated in a 5% CO_2_ chamber at 37 °C.

### In vivo xenograft studies

The present study was approved by the Committee of Care and Use of Laboratory Animals of Yonsei University College of Medicine. All animal experiments were performed according to the institutional guidelines and policies. This study was also performed in compliance with the ARRIVE guidelines.

The in vivo glutamine MRI experiment of this study largely consisted of two parts: MRI–IHC and MRI–ASCT2 inhibitor study. For the glutamine MRI–IHC study, 5-week-old male athymic nude mice (N = 5) were purchased from Orient Bio (Sungnam, Republic of Korea) and acclimated for 1 week. We established an in vivo xenograft model by suspending the HT29 cells in Matrigel (BD Bioscience) at a concentration of 2 × 10^6^ cells/200 μL, diluting them with DMEM at a 1:1 ratio, and injecting them into the lower part of the right flank of each mouse. Tumor sizes were measured every other day using calipers, and the tumor volumes were calculated using the following formula: tumor volume = the largest transverse diameter (width) × the largest longitudinal diameter (length) × (height/2). MRI was performed when the tumor volumes exceeded approximately 500 mm^3^. Following the MRI experiments, the mice were sacrificed, and the resected tumor masses were dissected. The dissected tumors were fixed in 4% paraformaldehyde and embedded in paraffin blocks for immunohistochemical staining.

For the glutamine MRI–ASCT2 inhibitor study, 6-week-old female athymic nude mice (N = 6) were purchased from Orient Bio and acclimated for 1 week. The ASCT2 inhibitor V-9302 (MedChemExpress, Monmouth Junction, NJ, USA) was reconstituted in a vehicle of phosphate-buffered saline supplemented with 2% dimethyl sulfoxide and administered intraperitoneally. The first MRI scans were performed 4 h after the treatment with the vehicle, and the second imaging experiments were performed 3 days later, 4 h after the treatment with V-9302 (75 mg/kg body weight). The experimental scheme is summarized in Fig. 1.

### MRI data acquisition

Prior to the imaging experiments, the mice were anesthetized with 1–2% isoflurane and a catheter was inserted in the tail vein to inject glutamine during the MRI. Anesthesia was maintained and the respiration of the mice was continuously recorded. Body temperature was maintained by circulating warm water. The MRI experiments were performed in a 9.4 T Bruker BioSpec scanner (Ettlingen, Germany) with a 40-mm transceiver volume coil. Glutamine uptake images were measured using the rapid acquisition with relaxation enhancement (RARE) protocol. In the MRI–IHC study, first, 30 pre-injection T_2_-weighted images were recorded. Approximately 200 µL of 200 mM glutamine solution (2 mmol/kg) was then manually injected intravenously over 20 s between scans 31 and 33, followed by the recording of 57 post-injection images (Fig. 1b). The imaging parameters were as follows: matrix = 240 × 120; field of view (FOV) = 36 mm × 18 mm; 5–13 slices in the axial direction, depending on the size of the tumor; slice thickness = 1 mm; RARE factor = 12 with effective echo time (TE) = 48 ms; and repetition time (TR) = 2000 ms. The total experimental time was 30 min. In the MRI-inhibitor study, T_2_-weighted images were recorded using the same pulse protocol. The imaging parameters were as follows: matrix = 192 × 96; effective TE = 48 ms; RARE factor = 12; and TR = 1875 ms. The number of pre- and post-injection images was adjusted to 20 and 60, respectively. Images were acquired every 15 s, and the total experimental time was 20 min.

### IHC staining

IHC staining was performed on 4-μm-thick sections obtained from paraffin-embedded xenograft tissues to determine the expression of GLS, glutamine transporters (ASCT2 and SNAT2), and CSC markers (CD44 and CD166). These results were compared with those from the glutamine uptake MRI. The paraffin-embedded sections were deparaffinized in xylene and rehydrated in gradually decreasing concentrations of ethanol. Antigen retrieval was performed using a sodium citrate buffer (10 mM, pH 6.0) for 3 min in a heated pressure cooker. After incubation with 3% hydrogen peroxide for 30 min to block endogenous peroxidase activity, the sections were incubated in a blocking reagent for 30 min at room temperature.

The sections were incubated overnight at 4 °C with anti-CD44 (1:100 dilution; eBioscience, San Diego, CA, USA), anti-CD166 (1:150 dilution; Leica Biosystems, Newcastle, UK), anti-GLS (1:100 dilution; Abcam, Waltham, MA, USA), anti-SNAT2 (1:100 dilution; Bioss Antibodies, Woburnm MA, USA), and anti-ASCT2 (1:200 dilution; Cell Signaling Technology, Danvers, MA, USA) and then incubated with secondary antibodies. The sections were treated using the reagents of the Vectastain ABC kit (Vector Laboratories, Burlingame, CA, USA) and immunostained with 3,3′-diaminobenzidine (DAB; Dako, Carpinteria, CA, USA). After counterstaining with hematoxylin, the sections were evaluated using light microscopy, and the expression of ASCT2, GLS, SNAT2, CD44, and CD166 was determined. The intensity of positive staining was determined in the fields coincident with those in the MRI images for each sample at 400× magnification. The intensity score was determined automatically based on DAB and hematoxylin staining and was designated as high, medium, low positive, or negative using the automated digital image analysis software ImageJ (U. S. National Institutes of Health, Bethesda, MD, USA). Each staining score was converted to a percentage value using an IHC profiler^20^.

### MRI data processing

All MRI data were processed using MATLAB (MathWorks, Natick, MA, USA). The T_2_-weighted images for the MRI–IHC study were realigned to the first image of the series. Each image matrix was then resized to 120 × 100 for clarity. The change in intensity following the injection of glutamine solution was calculated by comparing the pre- and post-injection images using the following equation:

1 $$\Delta S(t) = \frac{S(0) - S(t)}{{S(0)}},$$

where S(0) is the average intensity of the pre-injection images, and S(t) is the intensity of the post-injection images as a function of time. The images for the MRI–inhibitor study were also realigned and resized (128 × 96), and then the intensity change was calculated in the same manner using Eq. (1).

### Statistical analysis

The IHC staining intensity scores were calculated using an IHC profiler plugin in the ImageJ software package. Statistical analyses were carried out on the data of images of areas with high and low glutamine uptake using the non-parametric Wilcoxon test. Data with p values < 0.05 were considered statistically significant.

In the MRI–inhibitor study, the effect of the inhibitor was analyzed using the two-tailed paired *t*-tests for parameters derived from the vehicle *vs.* V-9302 injection MRI data. Data with p values < 0.05 were considered statistically significant.

## Supplementary Information

Supplementary Figures.
