# Supplementary material for: MRI assessment of glutamine uptake correlates with the distribution of glutamine transporters and cancer stem cell markers
Source: Sci Rep. 2022 Apr 1;12:5511. doi: 10.1038/s41598-022-09529-7 (PMC8975836; doi:10.1038/s41598-022-09529-7)

Supplementary Fig. 1. MRI images of glutamine uptake in the mouse xenograft model using HT29 cells. The average of the intensity change over the first 4 min post-injection is displayed. Only the intensity change in the tumor region is displayed.


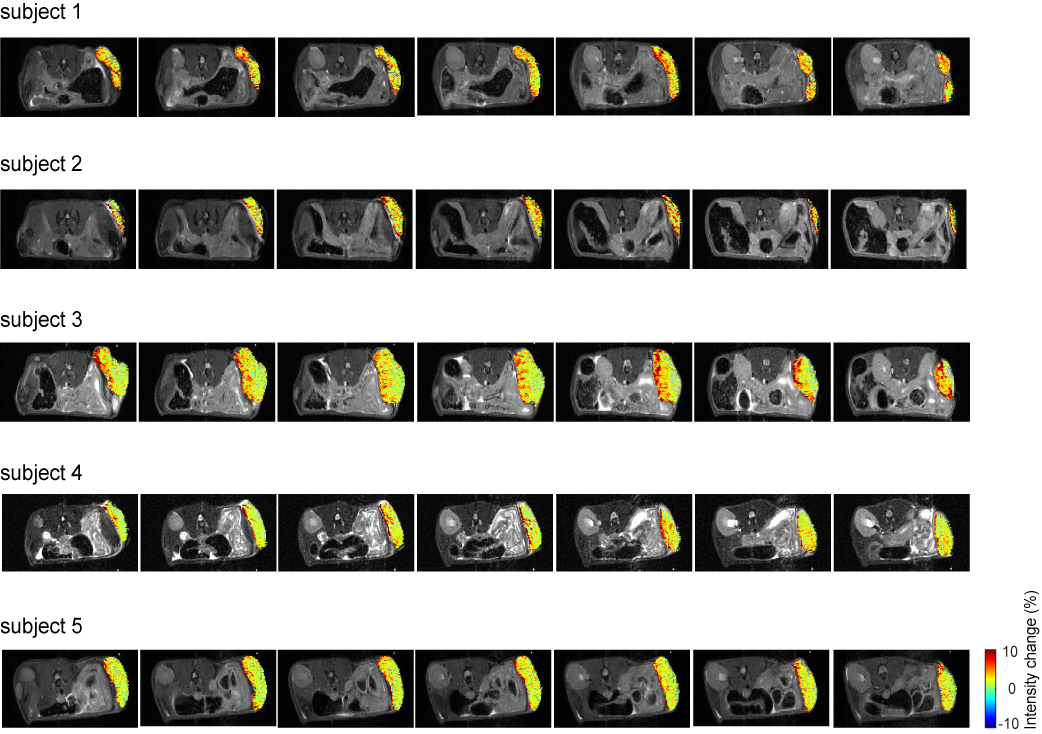


Supplementary Fig. 2. IHC of xenograft HT29 tumor in 5 subjects. (a) Regions with strong (marked in red) and weak (marked in blue) glutamine uptake were selected from MRI images, and compared with IHC in (b). The image of the tumor below shows IHC staining of ASCT2. Scale bar = 2 mm. IHC analysis of ASCT2, CD44, CD166, GLS and SNAT2 expression in tumor regions that show strong (top row, outlined in red) and weak (bottom row, outlined in blue) glutamine uptake. Scale bar = 50 μm.


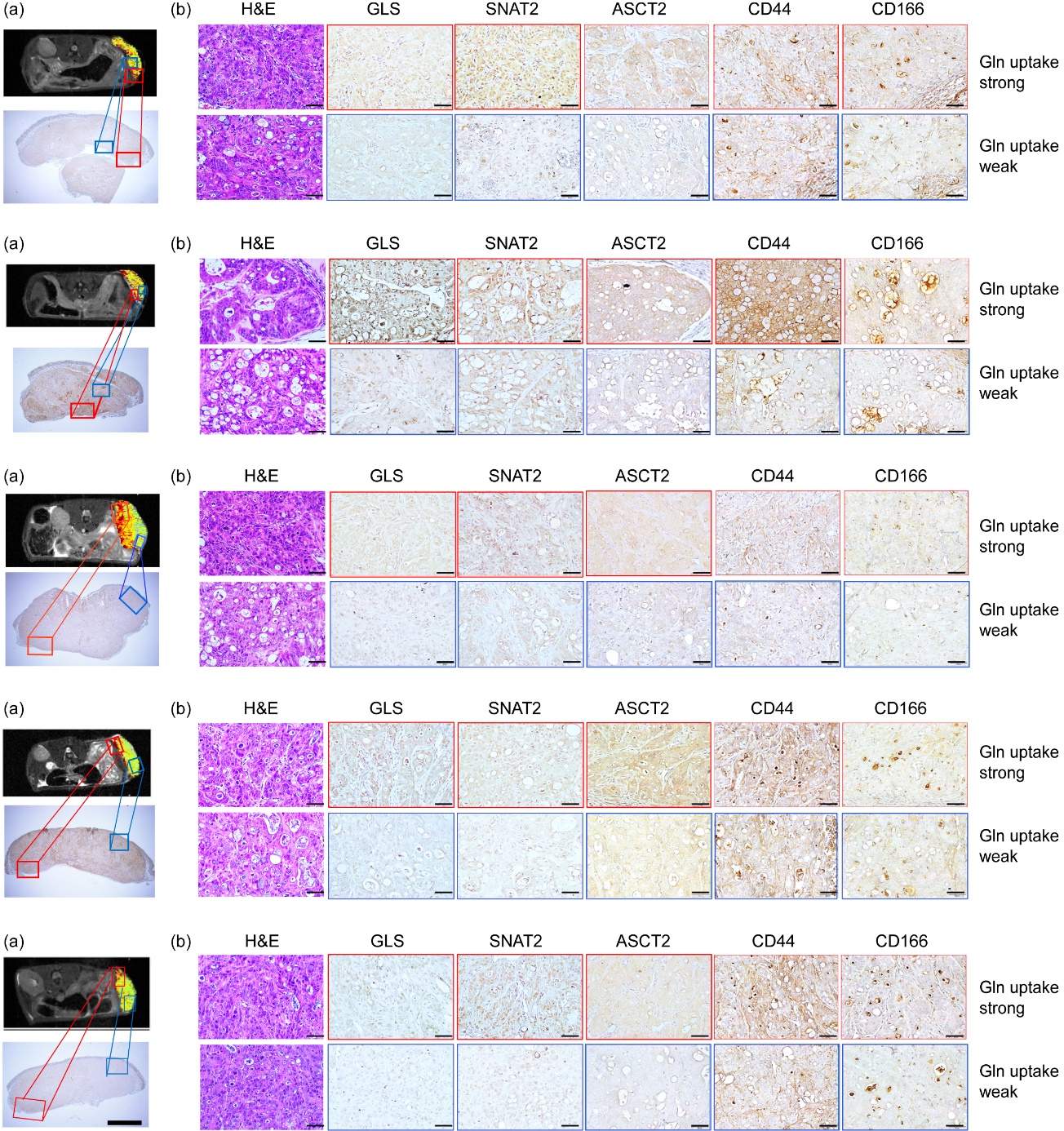


Supplementary Fig. 3. Comparison of the MRI intensity change of an individual mouse measured in vehicle- *vs*. V-9302-treated xenograft. (a) The sum of the intensity of pixels that show a change higher than 4% (s_int_) is plotted as a function of post-injection time. (b) Data in (a) were divided by the tumor size (a_tumor_) to account for the tumor growth during the 3 days between MRI scans.


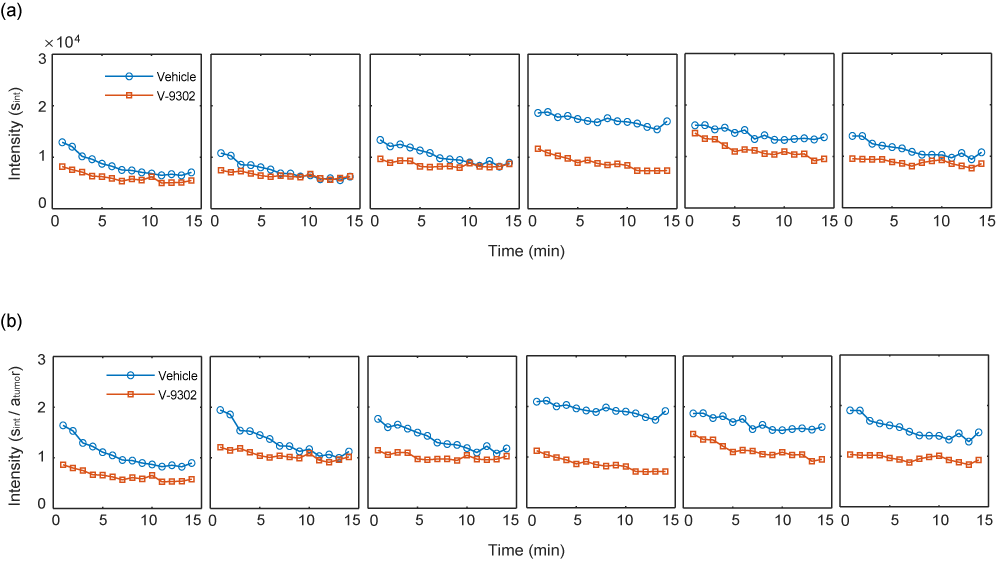

Supplement: Supplementary file 1 — Supplementary Figures. [file 41598_2022_9529_MOESM1_ESM.docx]
